# Predictors and time to poor management outcomes among pediatric patients hospitalized with pneumonia in the Gedeo Zone, southern Ethiopia: a prospective follow-up study

**DOI:** 10.3389/fped.2024.1447363

**Published:** 2025-01-17

**Authors:** Wagaye Alemu, Mebirat Ademassu, Firehiwot Belayneh, Yabibal Gebeyehu, Getachew Assefa Zenebe, Temesgen Leka Lerango

**Affiliations:** ^1^ School of Public Health, College of Health Sciences and Medicine, Dilla University, Dilla, Ethiopia; ^2^ Department of Midwifery, College of Health Sciences and Medicine, Dilla University, Dilla, Ethiopia; ^3^ Department of Pharmacy, College of Health Sciences and Medicine, Dilla University, Dilla, Ethiopia; ^4^ School of Medicine, College of Health Sciences and Medicine, Dilla University, Dilla, Ethiopia

**Keywords:** time, poor outcome, pneumonia, predictors, pediatrics, prospective, follow-up, Ethiopia

## Abstract

**Background:**

Pneumonia and other lower respiratory tract infections are the leading causes of death worldwide. Accurate diagnosis, identification of complications and underlying conditions, and appropriate treatment are essential for preventing pneumonia-related morbidity and mortality. Children in developing countries, such as Ethiopia, are at risk of contracting pneumonia, which could lead to death if not treated correctly. Therefore, we sought to assess the predictors and time to management outcomes among pediatric patients hospitalized with pneumonia in the Gedeo Zone, southern Ethiopia.

**Methods:**

A multicenter, institution-based prospective follow-up study was conducted among 484 pediatric patients hospitalized with pneumonia in the Gedeo Zone, southern Ethiopia. The data were entered into EpiInfo version 7 and exported to STATA version 15 for analysis. Survival analysis using a Cox proportional hazards model was performed to identify predictors of poor management outcomes. Associations between predictors and poor management outcomes were estimated using a *p*-value <0.05 and adjusted hazards ratios (AHR) with 95% CIs.

**Results:**

Among the 484 patients admitted with pneumonia, 381 (78.7%) recovered, 16 (3.3%) died, 6 (1.2%) were transferred out, and 81 (16.7%), defaulted. Over the study period, the incidence rate of poor management outcomes was 4 per 100 person-days of observation, while the incidence rate of recovery was 15 per 100 person-days of observation. According to the multivariable Cox regression analysis, the factors significantly associated with poor management outcomes were comorbidities at admission (AHR = 2.27, 95% CI: 1.01–5.26), age (AHR = 5.96, 95% CI: 2.71–13.1), nutritional status (AHR = 1.54, 95% CI: 1.08–3.17), and residence (AHR = 1.58, 95% CI: 1.05–2.34).

**Conclusion:**

The incidence rate of poor management outcomes was 4 per 100 person-days of observation. Comorbidities at admission, age, nutritional status, and place of residence were statistically significant predictors of poor management outcomes.

## Introduction

Community-acquired pneumonia (CAP) is an infectious disease affecting the lung parenchyma and adjacent organs. The primary causative agents are respiratory bacteria. On the other hand, as indicated in most studies, no specific pathogen could be identified in more than 50% of the cases (1). Streptococcus pneumoniae is the most commonly identified pathogen in all studies and across all settings [outpatients, inpatients, and intensive care unit (ICU) patients with CAP] (1–3). *Haemophilus influenzae* (HI) is also frequently detected in outpatients (13%), but its prevalence is much lower in hospitalized patients (6%–7%). Respiratory viruses constitute another important group of pathogens, affecting both outpatients and inpatients (1). Seasonal influenza viruses contribute to yearly increases in CAP incidence and lead to increased mortality in patients coinfected with bacterial pathogens (4, 5). Another relevant group is the so-called “atypical” bacteria. Among these, *Mycoplasma pneumoniae* is frequent in young patients with CAP (7%–12%) and usually follows a benign course (6, 7).

Viral, bacterial, and fungal infections constitute the most common causes of childhood pneumonia (8, 9). Childhood pneumonia is typically caused by respiratory syncytial virus, *S. pneumoniae*, and *H. influenzae*, with the latter two being preventable through vaccination (10). Severe pneumonia is diagnosed when children with an acute onset of cough and/or difficulty breathing present with any danger signs, such as central cyanosis, inability to breastfeed or drink, vomiting, convulsions, lethargy, unconsciousness, grunting, or head nodding (11).

Poor socioeconomic status, inability to breastfeed, malnutrition, indoor air pollution, household crowding, low birth weight, incomplete immunization schemes, HIV, prolonged illness duration, and underlying chronic illnesses, including heart disease, are some of the main risk factors for pediatric pneumonia (12–14). Almost all pneumonia deaths (98%) occur in developing countries, with Africa and Southeast Asia accounting for more than three-quarters of them (15). The World Health Organization’s (WHO) African region had the highest burden of severe pneumonia mortality (0.5 million) in 2015 (16). Despite advancements in prevention strategies, approximately 4 million cases of childhood pneumonia are reported every year in Ethiopia, making pneumonia one of the leading causes of morbidity and mortality among children (17). Despite the availability of adequate antimicrobial therapy, approximately 10% of all patients with CAP do not survive pulmonary infection, and mortality rates for severe CAP have plateaued at 20%–30% for the last few decades, especially in cases where treatment failure occurs (18). Investigating the management outcomes of pediatric patients hospitalized with pneumonia is crucial for evaluating the effectiveness of pneumonia care in healthcare settings. Therefore, this study aimed to investigate the management outcomes and their predictors among hospitalized pediatric patients in the Gedeo Zone, southern Ethiopia, using a prospective design that provides robust, recent, and more credible evidence for decision-makers and contributes to the scientific world.

## Methods and materials

### Study setting

**Figure 1:**
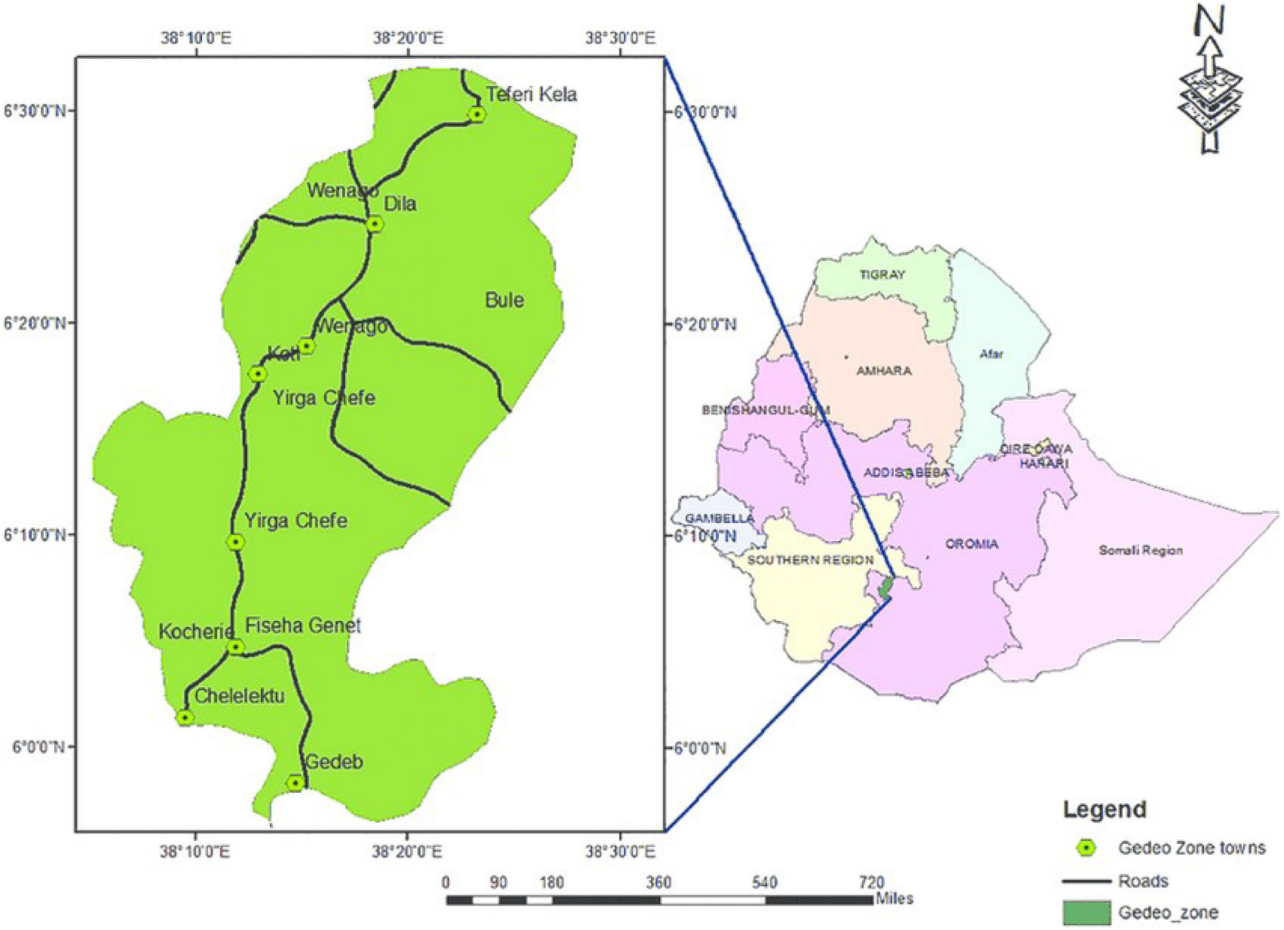
Map of the study area, southern Ethiopia and Gedeo Zone, with permission from the Gedeo Zone Administrative Office.

The study was conducted in four governmental hospitals in the Gedeo Zone, southern Ethiopia, from September 2021 to September 2022. The Gedeo zone comprises eight woredas and two city administrations. It is served by 38 health centers and 4 public hospitals (Figure 1).

### Study design

A multicenter, institution-based prospective follow-up study was conducted.

### Participants

The study participants included all pediatric patients hospitalized with pneumonia in the four public hospitals of the Gedeo Zone from September 2021 to September 2022. Patients aged ≤14 years who were admitted with a clinical diagnosis of community-acquired pneumonia in pediatric wards of hospitals in the Gedeo Zone were included. Patients with uncertain diagnoses of pneumonia, patients who died before treatment was initiated, and patients who were referred to other healthcare facilities were excluded.

### Sample size determination

To determine the sample size, various predictors significantly associated with the outcome variable were considered. Accordingly, the sample size was determined using a double population proportion formula by considering the percentage of unexposed individuals with the outcome (24.76%), ratio (1), power (80%), and 95% confidence level using living in a home with smokers as a predictor based on a study conducted in Rabat, Morocco (19). Using the above assumptions, the total sample size was calculated to be 474, and anticipating a 10% loss to follow-up, the final sample size was adjusted to 527.

### Sampling procedure

All public hospitals in the Gedeo Zone were selected. The final sample size was proportionally allocated to each hospital based on the number of pediatric patients hospitalized with pneumonia in the last year, which served as a benchmark. All children who were hospitalized in these public hospitals with pneumonia during the study period were enrolled in the study.

### Operational definitions

*Treatment outcome:* The treatment outcome was assessed after administering a drug, after management during admission, or after admission in the four public hospitals in the Gedeo Zone. The possible outcomes included recovered, death, transferred out, defaulted, and transferred to the ICU.

*A good treatment outcome* was defined as discharge from the hospital on the grounds of clinical improvement (20).

*A poor treatment outcome* was defined as the occurrence of death, complications requiring transfer to ICU, or default (20).

*Vaccination status:* According to the guidelines of the Ethiopian Ministry of Health, vaccinations for children under 5 years of age are provided as part of the Expanded Program on Immunization (EPI). The routine immunization schedule includes the following vaccines: BCG (Bacillus Calmette–Guérin), polio vaccine, pentavalent vaccine (DPT-HepB-Hib), pneumococcal conjugate vaccine (PCV), rotavirus vaccine, measles and rubella vaccine, vitamin A supplementation, and deworming.

*Fully vaccinated:* A child was considered fully vaccinated if they had received all the above-listed vaccines.

*Partially vaccinated:* A child was considered partially vaccinated if they had received only one of the above-listed vaccines.

*Not vaccinated:* A child was considered not vaccinated if they had not received any of the above-listed vaccines.

*Not known*: A child’s vaccination status was labeled as not known if no information could be obtained about the vaccination history of the child.

### Data collection procedures

Sociodemographic details of the participants were collected using a structured, interviewer-administered questionnaire. A standardized data extraction checklist was prepared by the investigators in English and translated into the local language. The data collection tool was developed to collect patients’ demographic and clinical characteristics including diagnostic method(s) used for disease identification, pertinent laboratory findings, comorbidities, medications administered, type and severity of the disease, discharge information (treatment outcome), and duration of hospital stay from the patient's medical charts, treatment charts, and laboratory data reports. Pneumonia was diagnosed through clinical assessment, complete blood count (CBC) laboratory tests, and chest x-rays. Comorbidities were assessed and determined based on clinical evaluations and laboratory findings performed by hospital clinicians. The data were collected by four trained data collectors.

### Data quality control

Training on the objectives of the study and how to review the documents as per the data extraction format was given to the data collectors and supervisors for 1 day before data collection. The data extraction checklist was pretested for consistency in understanding the review tools and completeness of the data items, and necessary amendments were made to the final data extraction format. The overall process was supervised by the principal investigator and supervisors assigned to the project. Daily checks were made by the principal investigator and/or supervisors to ensure that all the forms were completed correctly.

### Data processing and analysis

The data were entered into EpiInfo version 7, cleaned using SPSS version 20 software, and exported to STATA version 15 for analysis. Descriptive and summary statistics were performed. Chi-square cross-tabulation was conducted to check the proportion of the variable categories. Person-time at risk was measured starting from the initiation of treatment or management (at the time of admission or after admission) when the patient was diagnosed with severe community-acquired pneumonia (SCAP) based on clinical characteristics, laboratory findings, and x-rays (as determined by the physician in the four Gedeo Zone hospitals) until each patient ends the follow-up (death, recovered, defaulted, transferred out, and transferred to the ICU). The Schoenfeld residuals test and the -Ln(-ln) graph were used to check the Cox proportional hazards assumption, which was confirmed to be fulfilled; following this, survival analysis was performed using a Cox proportional hazards model to identify predictors of poor management outcomes. As this study was a follow-up study, the Cox proportional hazards models was better than logistic regression to consider the follow-up time. So, both bivariable and multivariable Cox proportional hazards models were fitted to identify predictor variables. Variables with a *p*-value ≤0.2 in the bivariable analysis were fitted into the multivariable model. The 95% confidence interval (CI) of the hazards ratio (HR) was computed, and variables with a *p*-value ≤0.05 in the multivariable Cox proportional hazards model was considered predictors. Finally, the result is presented in sentences, figures, and tables.

## Results

### Sociodemographic characteristics

Among the 484 children admitted with community-acquired pneumonia, almost half (53.7%) were boys, and approximately two-thirds (68.2%) of the participants were 12–60 months old. Half (50.2%) of the participants were from rural residences, and almost half (48.3%) of the caretakers had no formal education (Table 1).

**Table 1:** Sociodemographic characteristics of pediatric patients hospitalized with pneumonia in the Gedeo Zone, southern Ethiopia.

Variables	Category	Frequency	Percentage
Gender	Female	224	46.3
	Male	260	53.7
Residence	Urban	241	49.8
	Rural	243	50.2
Age (in months)	<12	154	56.4
	12–60	330	43.6
Caregiver's educational status	No formal education	234	48.3
	Primary	209	43.2
	Secondary and above	41	8.5
Caregiver's marital status	Not married	8	1.7
	Married	448	92.9
	Divorced	28	5.4
Breastfeeding	Yes	411	84.9
	No	73	15.1
Vaccination status	Fully vaccinated	113	24
	Partially vaccinated	243	51.7
	Not vaccinated	91	19.4
	Not known	37	49

### Clinical and treatment-related characteristics

The median (IQR) length of hospital stay for children admitted in the hospital was 5 (4–7) days. Two-thirds of them (67.8%) stayed in the hospital for 4–7 days. Most of the study participants (84.9%) were breastfed. Most of the study participants (87%) had comorbidities, with the most common comorbidities being acute gastroenteritis and childhood asthma. Almost half of the subjects (48.8%) were dehydrated at admission, of which 23.8%, 21.7%, and 4.8% had mild, moderate, and severe dehydration, respectively. Almost two-thirds of the study participants (64.5%) were diagnosed clinically and based on complete blood count, and almost all (96.1%) of them were newly admitted (Table 2).

**Table 2:** Clinical characteristics of pediatric patients hospitalized with pneumonia in the Gedeo Zone, southern Ethiopia.

Variables	Category	Frequency	Percentage
Comorbidity at admission	Yes	421	87
	No	63	13
Type of comorbidity	AGE	146	34.3
	TB	19	4.5
	Childhood asthma	33	7.7
	More than one	203	47.7
	Other	25	5.2
Diagnosis	CBC and clinical assessment	314	64.9
	Chest x-ray	58	11.98
	CBC and x-ray	112	23.1
Main diagnosis (outcomes)	Recovered	381	78.7
	Died	16	3.3
	Transferred out	6	1.2
	Defaulted	81	16.7
Length of hospital stay (in days)	≤3	113	23.3
	>3	371	76.7
Consciousness at admission	Conscious	439	90.7
	Impaired	45	9.3
Nutritional status	Malnourished	70	16.7
	Not malnourished	348	83.3
Anemic status	Yes	154	31.8
	No	330	68.2
Admission status	New admission	465	96.1
	Readmission	9	1.9
	Referred from other	10	2.1

### Outcome status of pneumonia patients

**Figure 2:**
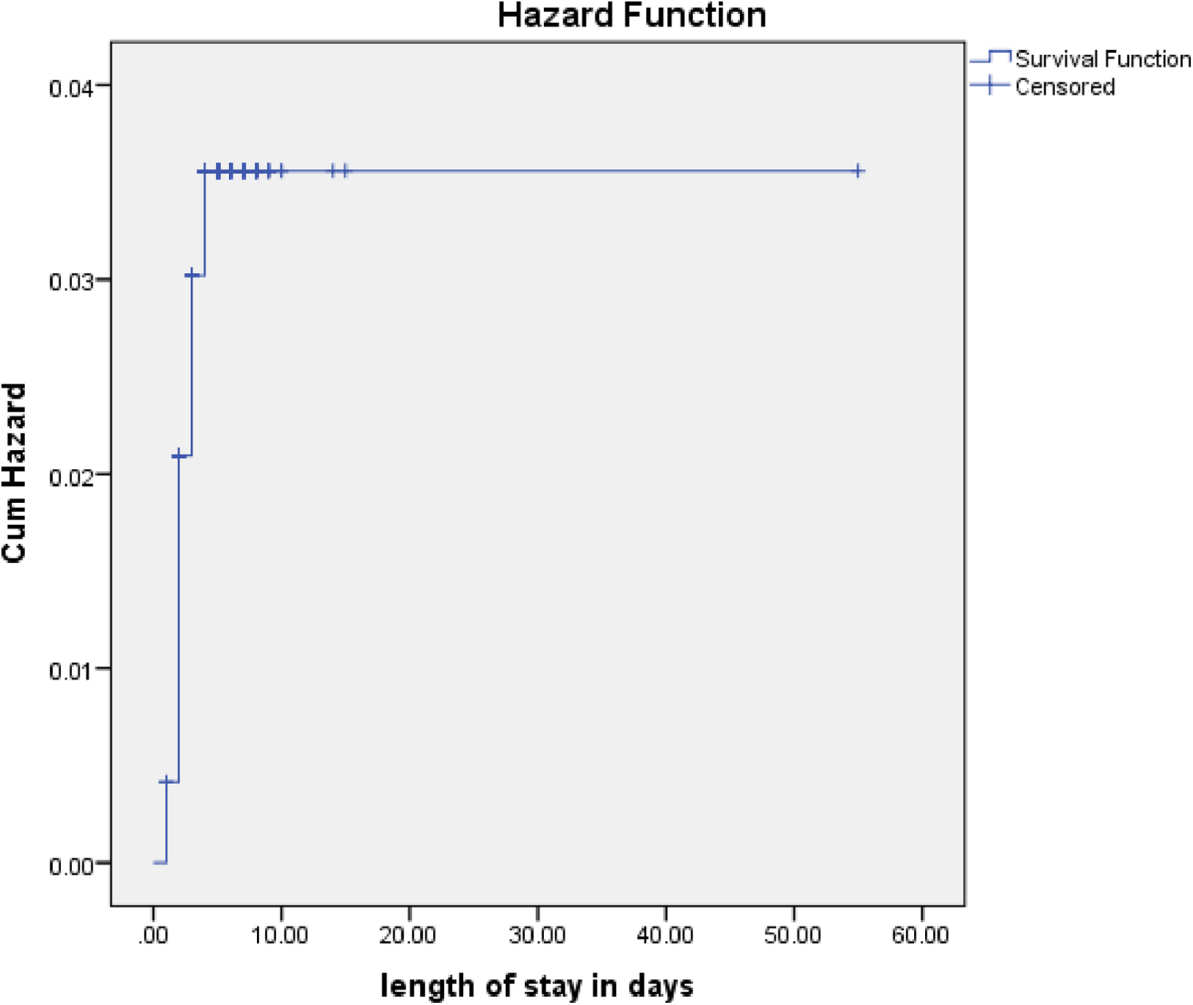
Cumulative hazards rate for poor management outcomes among pediatric patients hospitalized with pneumonia.

Among the 484 patients admitted with pneumonia, 381 (78.7%) recovered, 16 (3.3%) died, 6 (1.2%) were transferred out, and 81 (16.7%) defaulted. The study cohort included a total of 2,530 person-days of observations. Over the study period, the incidence rate of poor outcomes was 4 per 100 person-days of observation, while the incidence rate of recovery was 15 per 100 person-days of observation (Figure 2).

### Predictors of poor management outcomes

In the bivariable Cox proportional hazards analysis, family educational status, residence, comorbidities during admission, age of the child, consciousness status, nutritional status (mid-upper arm circumference, MUAC) at admission, and length of stay in the hospital were found to be candidates for multivariable analysis, with *p*-values <0.2. However, in the multivariable Cox regression analysis, comorbidities during admission, age, nutritional status, and residence were independent predictors of poor management outcomes among children admitted with pneumonia (Table 3).

**Table 3:** Bivariable and multivariable Cox regression for predictors of poor management outcomes among pediatric patients hospitalized with pneumonia in the Gedeo Zone, southern Ethiopia.

Variables	Category	Poor management outcomes		CHR (95% CI)	AHR (95% CI)
		Censored	Event		
Caregiver's educ. status	No formal education	178	56	1.54 (1.12–2.13)	1.33 (0.89–1.99)
	Primary and above	203	47	1	1
Age (in months)	>12	212	81	2.26 (1.41–3.62)	5.96 (2.71–13.1)*
	≤12	169	22	1	1
Residence	Rural	181	62	1.65 (1.11–2.45)	1.58 (1.05–2.34)*
	Urban	200	41	1	1
Consciousness	Impaired	32	13	2.37 (1.08–5.2)	1.14 (0.79–1.64)
	Conscious	349	90	1	1
Length of hospital stay (in days)	≤3	49	64	1.7 (1.44–3.44)	1.29 (0.2–1.39)
	>3	332	39	1	1
Comorbid at admission	Yes	48	12	1.94 (1.11–4.12)	2.27 (1.02–5.26)*
	No	333	91	1	1
Nutritional status	Malnourished	46	24	1.64 (1.17–2.27)	1.54 (1.08–2.17)^a^
	Not malnourished	285	63	1	1

CHR: crude hazard ratio; AHR, adjusted hazard ratio.

* Significant predictor at *p* < 0.05.

**Figure 3:**
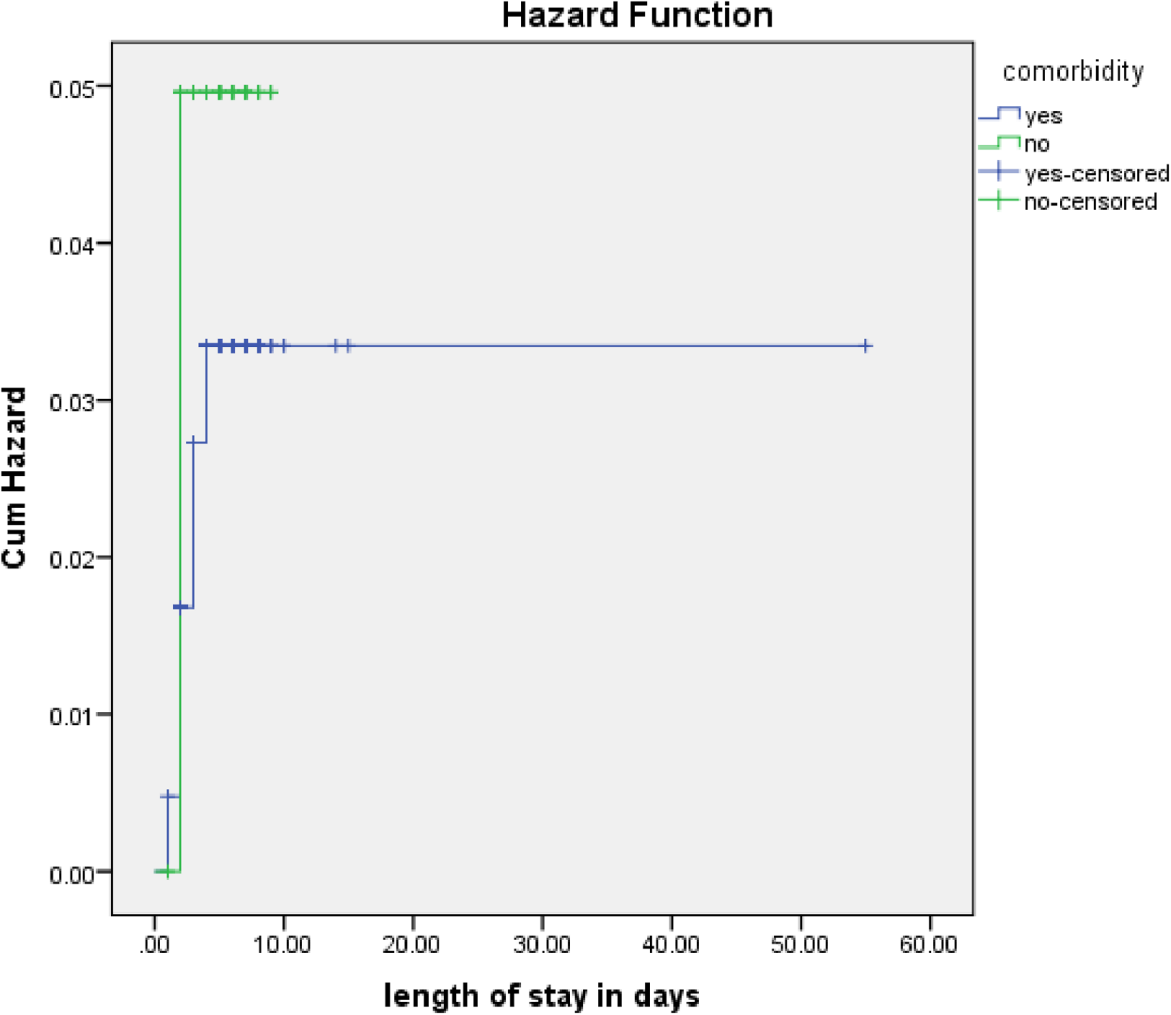
Estimated cumulative hazards rate for comorbidities at admission.

**Figure 4:**
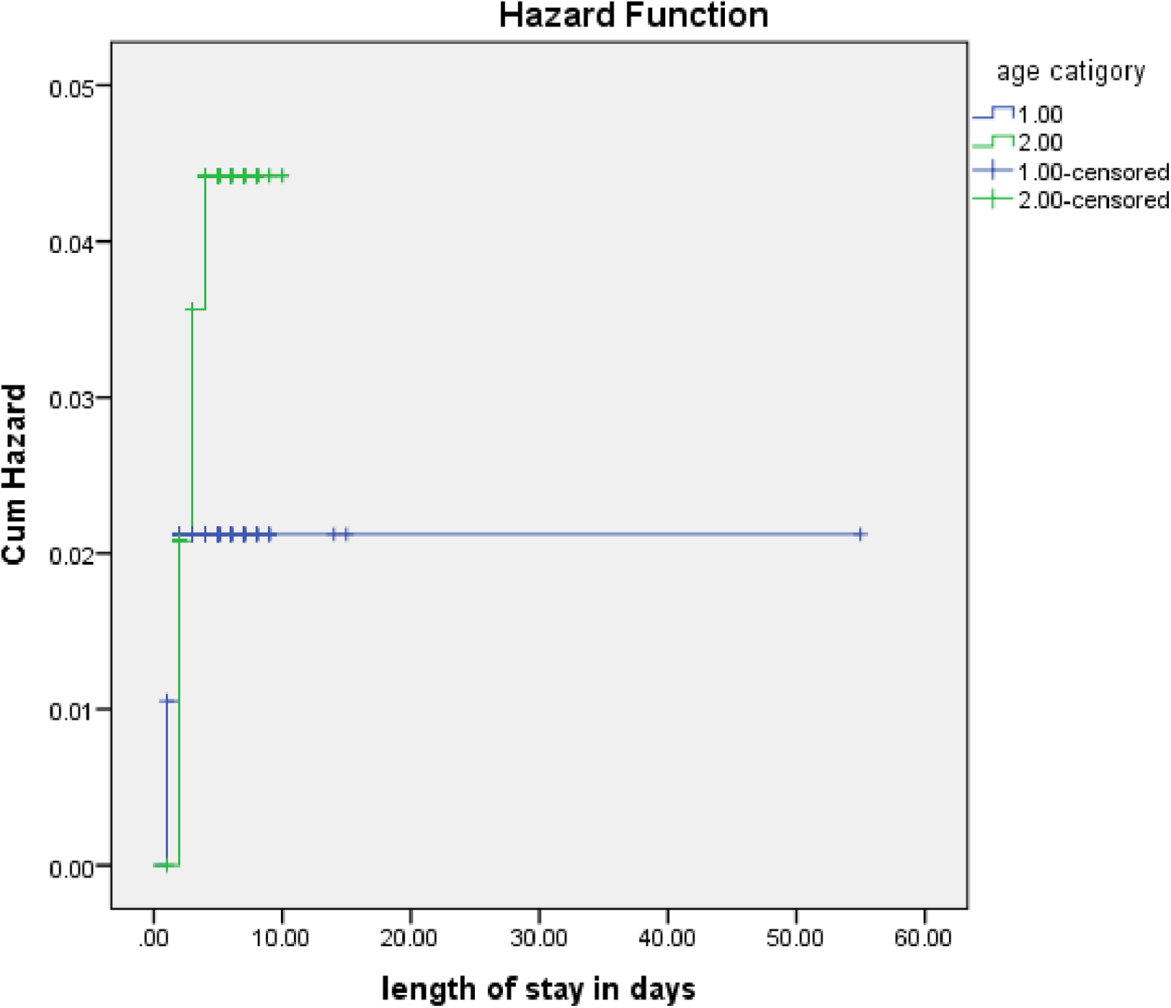
Estimated cumulative hazards rates for different age categories of children.

**Figure 5:**
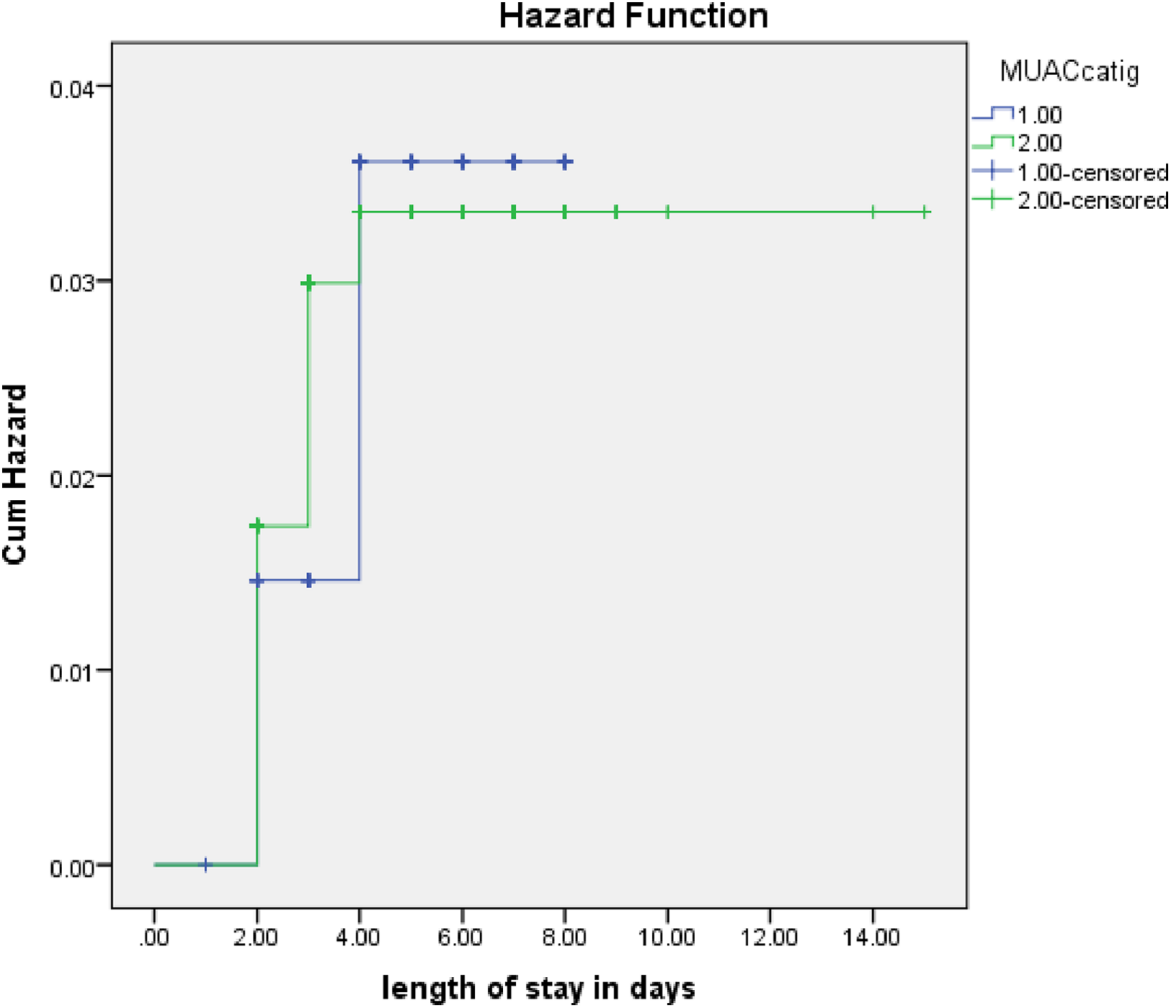
Estimated cumulative hazards rate for different nutritional statuses at admission.

**Figure 6:**
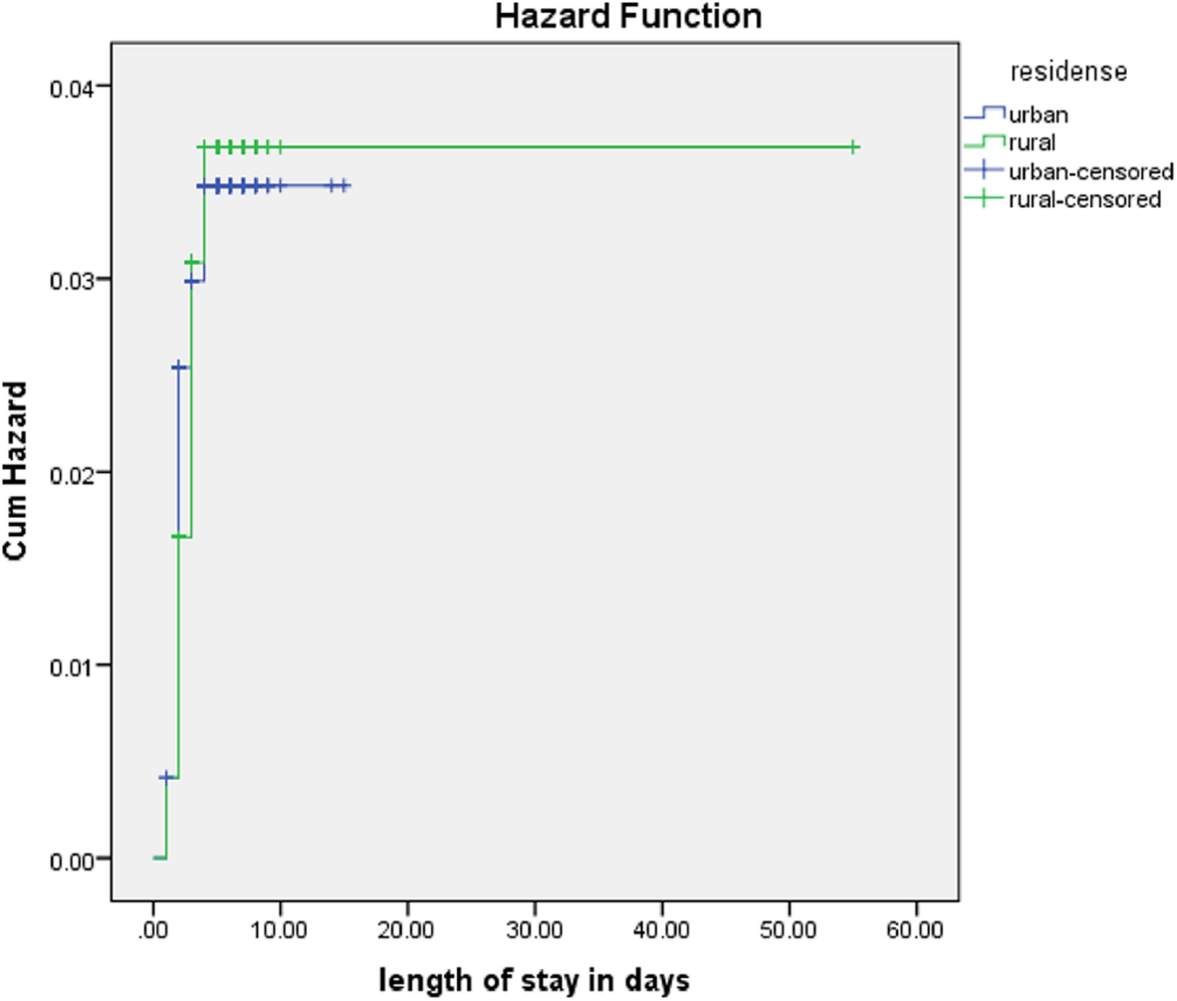
Estimated cumulative hazards rates for different residences.

The rate of poor management outcomes was 2.27 times greater among patients who had comorbidities during admission compared to those without [adjusted hazards ratios (AHR) = 2.27, 95% CI: 1.01–5.26] (Figure 3). The risk of poor management outcomes was 6 times greater among patients aged 12 months or older than among those aged 12 months or younger (AHR = 5.96, 95% CI: 2.71–13.1) (Figure 4). Malnourished patients were 1.54 times more likely to have poor management outcomes than those who were not malnourished (AHR = 1.54, 95% CI: 1.08–3.17) (Figure 5). The risk of poor management outcomes was 1.6 times greater among patients from rural residences compared to those from urban residences (AHR = 1.58, 95% CI: 1.05–2.34) (Figure 6).

## Discussion

This study aimed to assess the predictors and time to management outcomes among pediatric patients hospitalized with pneumonia. The incidence rate of poor management outcomes in this study was 4 per 100 person-days of observation, while the incidence of recovery was 15 per 100 person-days of observation. Comorbidities at admission, age, nutritional status, and residence were found to be significant predictors of poor management outcomes in the study.

The incidence rate of poor management outcomes in this study is higher than that reported in a study conducted at the University of Gondar Specialized Hospital (13.5 per 100 person-days of observation) (21) but lower than that reported in a study conducted at Debre Markos University Specialized Hospital (16.25 per 100 person-days of observation) (22) and a study conducted at Nigist Eleni Mohammed Memorial Comprehensive Specialized Hospital, Hossana, Ethiopia (24.16 per 100 person-days of observation) (23). In this study, 3.3% of the children admitted with severe community-acquired pneumonia died, which is similar to a study done in Morocco, wherein 4% of patients died (19). However, the proportion of mortality in this study is lower than that in a study performed in India (10.5%) (24) and Jimma University Specialized Hospital (20.2%) (25), but it is higher than that in a study performed at Debre Markos University Specialized Hospital (2.27%) (22). In this study, the overall poor management outcome was 20% (95% CI: 17.8–25).

In the present study, the median (IQR) length of hospital stay was 5 (4–7) days, which is in line with a study done in Debre Markos reporting 4 (3–7) days (22), while it is lower than that in a study conducted at Jimma University Specialized Hospital (11.49 days) (25). The reason for these differences might be attributed to variations in the study settings, study design, follow-up time, sample size, and socioeconomic status of the study participants.

In this study, different factors were identified as predictors of poor management outcomes. In this study, having comorbidities at admission emerged as a significant predictor. Children admitted with comorbidities had poorer management outcomes than those admitted without comorbidities. This finding is in line with other studies (22, 25). This might be because when children suffer from multiple illnesses simultaneously, their immune systems are significantly compromised, which might lead to poor outcomes.

Age of the child was also a predictor variable in this study. According to the findings, older children were at greater risk of poor management outcomes than younger children. This finding is in line with other studies (21, 23, 25). In contrast, a similar study conducted in Nepal showed that older children recovered better than younger children (26). This discrepancy might be due to differences in the age groups of the children, as the studies included children under 6 and 3 years of age. Another possible reason for such contradiction could be a result of variations in baseline clinical conditions and treatment protocols across the study areas.

Nutritional status was identified as a statistically significant predictor in this study, which is similar to the findings of other studies (21, 23). Compared with normal children, malnourished children recovered more slowly. This is because malnutrition magnifies the severity of the disease and might lead to complications and increased time spent with illness (27). Undernutrition affects the immune system, consequently worsening the prognosis of the disease and resulting in difficult recovery (28).

Residence was also a predictor variable in this study. The reason for this might be that patients from rural areas might arrive at a delayed time to the hospitals to seek care, which can increase the severity of the disease. This finding contrasts another study, wherein in-hospital mortality rates, after adjusting for admission severity, were comparable or better for rural patients than for urban patients (29).

### Strengths and limitations of the study

The main strength of this study is the optimal sample size, and the follow-up time was adequate to estimate the management outcomes and their predictors. As a limitation, as this study was a follow-up study rather than an experimental study, it might show the temporal relationship but could not confirm the causal relationship between the outcome and predictor variables. In this study, only a few laboratory tests were done, but not all necessary laboratory tests were performed to give more soundable results. As the onset date of severe community-acquired pneumonia before arriving at the four hospitals was not captured, this might underestimate the total follow-up time.

## Conclusions

The incidence rate of poor management outcomes was higher in this study. Comorbidities at admission, age of the child, nutritional status, and residence were found to be significant predictors of poor management outcomes. Special attention should be given to improving the nutritional status of children. Comorbidities should be treated earlier, and special attention should be given to children with comorbidities.

## Data Availability

The raw data supporting the conclusions of this article will be made available by the authors without undue reservation.
